# Probiotic *Propionibacterium freudenreichii* requires SlpB protein to mitigate mucositis induced by chemotherapy

**DOI:** 10.18632/oncotarget.27319

**Published:** 2019-12-31

**Authors:** Fillipe Luiz Rosa do Carmo, Houem Rabah, Barbara Fernandes Cordeiro, Sara Heloisa da Silva, Rafaela Miranda Pessoa, Simone Odília Antunes Fernandes, Valbert Nascimento Cardoso, Valérie Gagnaire, Martine Deplanche, Bruna Savassi, Alessandra Figueiroa, Emiliano Rosa Oliveira, Caio César Fonseca, Maria Izabel Alves Queiroz, Núbia Morais Rodrigues, Sávio Henrique de Cicco Sandes, Álvaro Cantini Nunes, Luisa Lemos, Juliana de Lima Alves, Ana Maria Caetano Faria, Ênio Ferreira, Yves Le Loir, Gwénaël Jan, Vasco Azevedo

**Affiliations:** ^1^Instituto de Ciências Biológicas, Universidade Federal de Minas Gerais (UFMG), Belo Horizonte, Minas Gerais, Brasil; ^2^STLO, INRA, Agrocampus Ouest, UMR1253, Science & Technologie du Lait & de l'Oeuf, Rennes, France; ^3^Bba, Pôle Agronomique Ouest, Régions Bretagne et Pays de la Loire, Rennes, France; ^4^Department of Clinical and Toxicological Analysis, Federal University of Minas Gerais (UFMG), Belo Horizonte, Minas Gerais, Brazil

**Keywords:** mucositis, probiotic, surface protein, immunomodulation, inflammation

## Abstract

*Propionibacterium freudenreichii* CIRM-BIA 129 (*P. freudenreichii* wild type, WT) is a probiotic bacterium, which exerts immunomodulatory effects. This strain possesses extractable surface proteins, including SlpB, which are involved in anti-inflammatory effect and in adhesion to epithelial cells. We decided to investigate the impact of *slpB* gene mutation on immunomodulation *in vitro* and *in vivo*. In an *in vitro* assay, *P. freudenreichii* WT reduced expression of IL-8 (p<0.0001) and TNF-α (p<0.0001) cytokines in LPS-stimulated HT-29 cells. *P. freudenreichii* Δ*slpB*, lacking the SlpB protein, failed to do so. Subsequently, both strains were investigated *in vivo* in a 5-FU-induced mucositis mice model. Mucositis is a common side effect of cytotoxic chemotherapy with 5-FU, characterized by mucosal injury, inflammation, diarrhea, and weight loss. The WT strain prevented weight loss, reduced inflammation and consequently histopathological scores. Furthermore, it regulated key markers, including Claudin-1 *(cld1*, p<0.0005) and IL-17a (*Il17a*, p<0.0001) genes, as well as IL-12 (p<0.0001) and IL-1β (p<0.0429) cytokines levels. Mutant strain displayed opposite regulatory effect on *cld1* expression and on IL-12 levels. This work emphasizes the importance of SlpB in *P. freudenreichii* ability to reduce mucositis inflammation. It opens perspectives for the development of probiotic products to decrease side effects of chemotherapy using GRAS bacteria with immunomodulatory surface protein properties.

## INTRODUCTION

*Propionibacterium freudenreichii* represents the main species of dairy propionibacteria. It is a gram-positive, non-motile, non-spore forming and anaerobic to aerotolerant beneficial bacterium, which plays an important role in food transformation, particularly in cheese ripening [1]. It has been listed in the Qualified Presumption of Safety list by the European food safety authority [2]. It was given the GRAS (Generally Recognized As Safe) status for its use in cheese [3]. Dairy propionibacteria are peculiar bacteria with a great probiotic potential. They produce the short chain fatty acids (SCFAs) acetate and propionate, and other beneficial metabolites, such as vitamin B9 and B12, as well as 1,4-dihydroxy-2-naphthoic acid (DHNA) and 2-amino-3-carboxy-1,4-naphthoquinone (ACNQ), which were described as bifidogenic growth stimulators [1].

Probiotic effects of *P. freudenreichii* also include modulating the gut microbiota and the gut immune system [2]. In 2012, Cousin and collaborators demonstrated that dairy propionibacteria induce production of the regulatory cytokine IL-10 *ex vivo* in porcine colonic mucosa explants, and decrease production of proinflammatory cytokines, such as IL-8 and tumor necrosis factor-α (TNFα), in the gut mucosa of piglets after lipopolysaccharides (LPS) stimulation [4].

*P. freudenreichii* strains, isolated or associated with other probiotic bacteria, have also been shown to attenuate colitis induced by trinitrobenzene sulfonic acid (TNBS), in BALB/c mice [5]. *P. freudenreichii* was also reported to reduce intestinal and systemic proinflammatory alterations, caused by a high-fat diet, in a mice model [6]. Moreover, dairy propionibacteria strains may alleviate symptoms and stabilize the intestinal microbiota in patients with irritable bowel syndrome [7]. Altogether, these studies attracted attention on *P. freudenreichii* as a promising probiotic to potentiate the treatment of inflammatory diseases [1].

*P. freudenreichii* strain ITGP20, equivalent to CIRM-BIA 129 (*P. freudenreichii* wild type, WT), was used for the development of two experimental cheeses, one single-strain, and one in association with *Lactobacillus delbrueckii* subsp *lactis* CNRZ327. Both cheeses gave promising results and alleviated TNBS-induced colitis in mice [8, 9]. *P. freudenreichii* anti-inflammatory effect was further shown to depend on specific extractable surface proteins [10]. A surface proteomic analysis of *P. freudenreichii* extractable surface proteins identified three surface-exposed ones, designated SlpA, SlpB and SlpE [10]. Interestingly, the extraction of surface proteins from *P. freudenreichii* WT by guanidine hydrochloride suppresses its ability to induce anti-inflammatory cytokines in human PBMCs [10]. Moreover, in *P. freudenreichii* WT, Carmo and collaborators confirmed that the surface protein SlpB is involved in adhesion to cultured human intestinal epithelial cells HT-29 [11], and mutation of the *slpB* gene caused drastic changes in surface properties [12]. In this context, the great probiotic potential of *P. freudenreichii* in the context of inflammatory bowel diseases [8, 9], and the presence of a characterized extractable surface protein SlpB with immunomodulatory activity [13] led us to challenge this bacterium in another animal model involving inflammation: chemotherapy-induced mucositis [14].

Mucositis is a severe inflammation that affects the Alimentary Tract (AT) of individuals undergoing cancer treatment based on radiotherapy or chemotherapy, such as 5-Flourouracil (5-FU) [15]. Disease is characterized by pathological changes in the small bowel. This includes the presence of degenerative enterocytes, leukocyte infiltrate in the lamina propria, increased mucus production and degeneration of goblet cells, atrophy of villi, hypoplasia and apoptosis of intestinal crypts [16–18]. The side effects are characterized by mucosal injury, inflammation, diarrhea, and weight loss. The currently available treatments of mucositis (cryotherapy, growth factors, anti-inflammatory and antimicrobial agents) are poorly effective and may not be well tolerated. In this context, some studies have proposed the use of probiotic bacterial strains, as promising candidates in the treatment or prevention of inflammatory conditions such as mucositis [14, 19]. Clinical studies indicate a positive effect of selected lactobacilli in patients with mucositis [20, 21], while nothing is known about the effect of probiotic propionibacteria. Accordingly, the MASCC/ISOO (Association of Supportive Care in Cancer/International Society of Oral Oncology) clinical practice guidelines for the management of mucositis secondary to cancer therapy [22] recently added new guidelines, including one suggestion for probiotic agents containing *Lactobacillus* species for the prevention of chemotherapy and radiation-induced diarrhea in patients with pelvic malignancy as an adjuvant treatment. This comes in addition with the previous guidelines in favor of amifostine, octreotide, sucralfate enemas and sulfasalazine.

The aim of this study is to evaluate the probiotic ability of *P. freudenreichii* CIRM-BIA 129 to protect mice against inflammatory mucositis damages induced by 5-FU, and to further investigate the impact of *slpB* gene mutation on such a protection.

## RESULTS

### *Propionibacterium freudenreichii* WT, yet not the *P. freudenreichii* Δ*slpB* mutant, prevents LPS-induced inflammation in HT-29 cells

**Figure 1:**
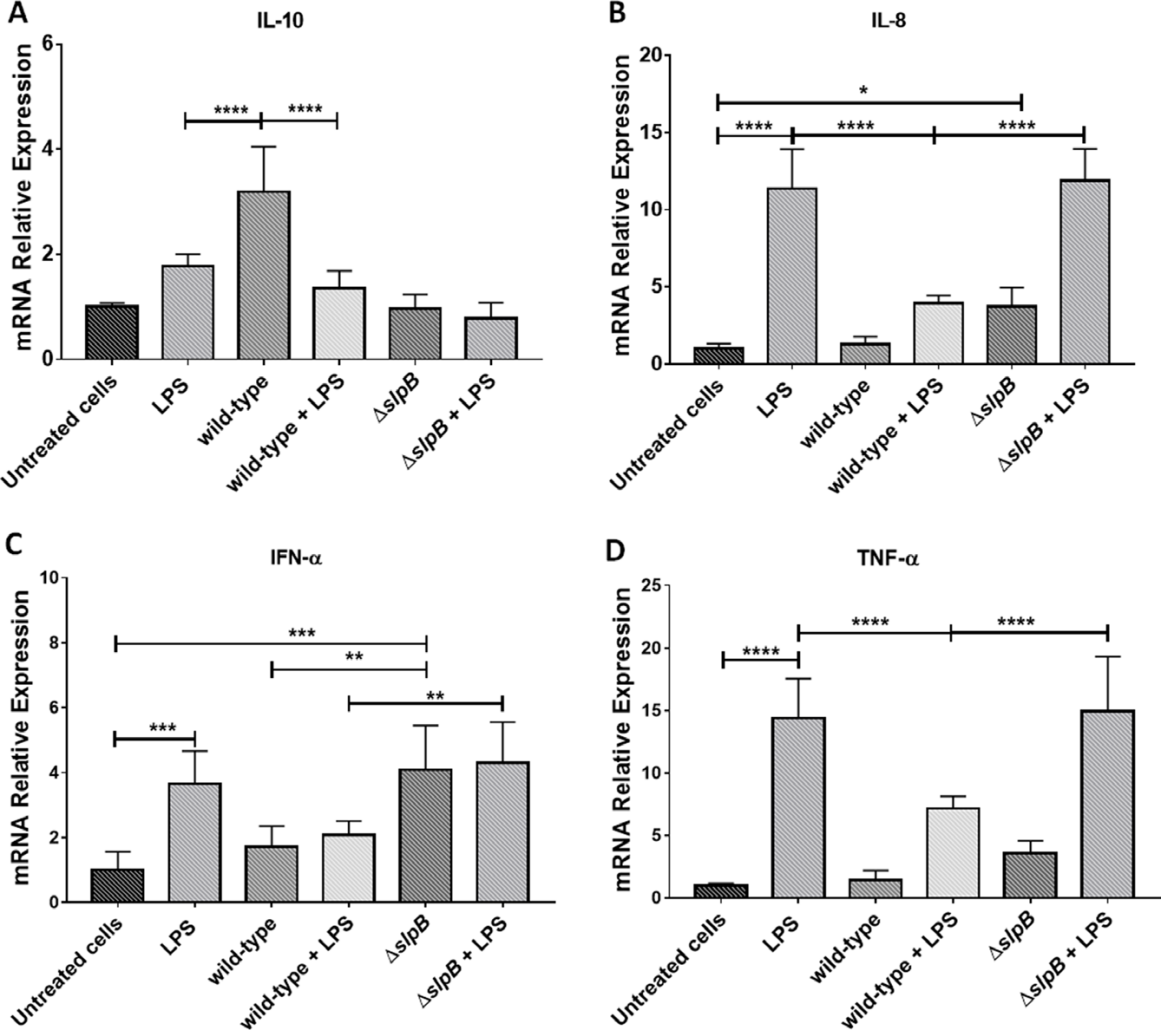
*Propionibacterium freudenreichii* Δ*slpB* mutant strain induces expression of pro-inflammatory cytokines in HT-29 cells Relative expression of cytokine genes encoding IL-10 **(A)**, IL-8 **(B)**, TNF-α **(C)** and IFN-α **(D)**, in HT-29 cells, stimulated by lipopolysaccharides (LPS), *P. freudenreichii* 129 WT, *P. freudenreichii* 129Δ*slpB*, or combinations thereof, was monitored by RT-PCR. Each cell treatment was done on 3 independent cultures (biological triplicates). Each quantification was done in triplicate (technical triplicates). The means and standard deviations are thus calculated from 9 values. Asterisks represent statistically significant differences between strains and were indicated as follows: *p < 0.05; **p < 0.01; ***p < 0.001, and ****p < 0.0001.

We investigated the anti-inflammatory potential of *P. freudenreichii* WT, and the impact of the mutation of the *slpB* gene on this potential. HT-29 cells, both in the presence and in the absence of proinflammatory Lipopolysaccharide (LPS) from *E. coli*, were exposed to both strains, WT and mutant. We monitored changes in the relative expression of genes involved in the inflammatory process (Figure 1).

*P. freudenreichii* WT induced expression of *il10* (Figure 1A), with significant differences, (*p < 0.0001*), compared to control non-treated cells. The mutant *P. freudenreichii* Δ*slpB* failed to induce *il10* expression, by contrast with the WT strain. LPS did not change *il10* expression, with or without co-stimulation with *P. freudenreichii* WT or *P. freudenreichii* Δ*slpB*. LPS strongly induced *il8* (Figure 1B). This induction was inhibited by the presence of *P. freudenreichii* WT, with a significant difference with LPS alone (p < 0.0001), yet not by the mutant *P. freudenreichii* Δ*slpB*. As a control, *P. freudenreichii* WT did not induce *il8* expression, while the mutant *P. freudenreichii* Δ*slpB* did, when compared to *P. freudenreichii* WT or to untreated control. Accordingly, both LPS and mutant *P. freudenreichii* Δ*slpB* induced *ifna* (p<0.001), while *P. freudenreichii* WT did not (Figure 1C). After LPS stimulus, *ifna* expression was higher in the presence of the mutant than in the presence of the WT (p<0.01). The pro-inflammatory *tnfa* was induced by LPS, yet not by *P. freudenreichii*, neither WT, nor mutant (Figure 1D). The WT repressed LPS-mediated induction of *tnfa*, while the mutant did not.

**Figure 2:**
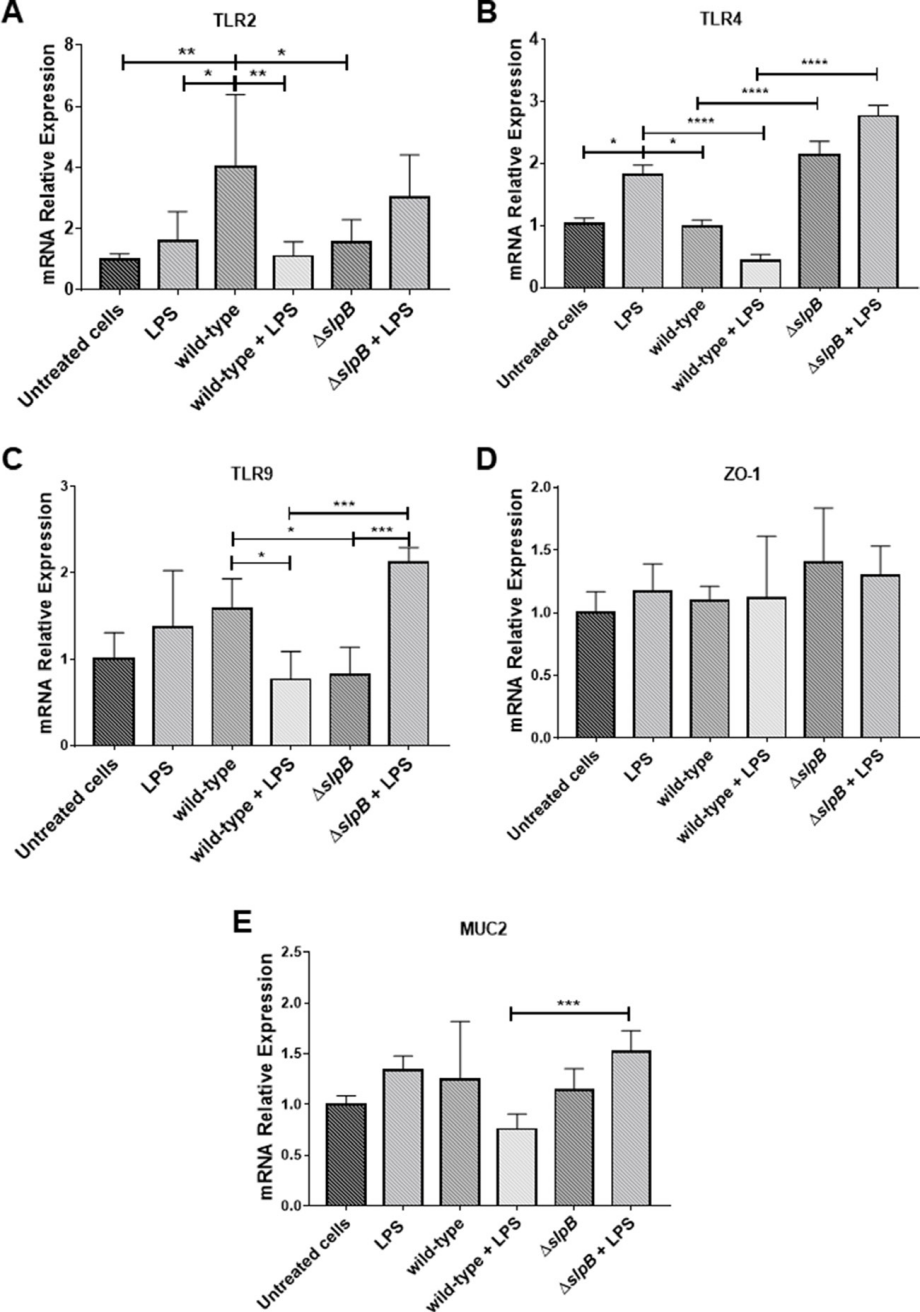
*Propionibacterium freudenreichii* WT strain modulates *in vitro* expression of in Toll-like receptors (TLRs) in HT-29 cells Relative expression of genes encoding TLR2 **(A)**, TLR4 **(B)**, TLR9 **(C)**, ZO1 **(D)** and of MUC2 **(E)**, in HT-29 cells stimulated by lipopolysaccharides (LPS), *P. freudenreichii* 129 WT, *P. freudenreichii* 129Δ*slpB*, or combinations thereof, was monitored by RT-PCR. Each cell treatment was done on 3 independent cultures (biological triplicates). Each quantification was done in triplicate (technical triplicates). The means and standard deviations are thus calculated from 9 values. Asterisks represent statistically significant differences between strains and were indicated as follows: *p < 0.05; **p < 0.01; ***p < 0.001, and ****p < 0.0001.

We then monitored expression of TLR2, TLR4 and TLR9 receptors genes. LPS *per se* had no effect on *tlr2* expression (Figure 2A). Contrastingly, *P. freudenreichii* WT induced expression of *tlr2*, in comparison with untreated cells (p < 0.01). LPS completely suppressed this *tlr2* induction (p < 0.01). The mutant *P. freudenreichii* Δ*slpB* had no significant effect on *tlr2* expression.

Concerning *tlr4* receptor gene expression (Figure 2B), it was induced by LPS, compared to the untreated control (p<0.05). The mutant strain also triggered *tlr4* expression significantly, compared to control (p<0.0021), while *P. freudenreichii* WT did not. This WT strain repressed LPS-induced expression of *tlr4* (p<0.0001), while the mutant strain lost this ability.

Neither LPS, nor *P. freudenreichii* WT modified *tlr9* expression significantly, compared to the control (Figure 2C). In control conditions, *tlr9* expression was significantly lower in the presence of the mutant than in the presence of the WT. In LPS-inflamed cells, the opposite was observed with a higher expression in the presence of the mutant than in the presence of the WT (p<0.001).

Regarding tight junction gene *zo1* (Figure 2D) we did not find any significant differences between controls untreated cells and stimulated cells. In LPS-inflamed cells, *muc2* (Figure 2E) was more expressed in the presence of the mutant than in the presence of the WT (p<0.0005), while none of these strains affected its expression in control conditions.

We then extracted surface extractable proteins from *P. freudenreichii* WT using guanidine hydrochloride and purified the SlpB protein following diafiltration and size-exclusion chromatography of this extract. The purified protein was then used to stimulate cultivated HT-29 cells. As shown in Supplementary Figure 1, the purified SlpB protein induced *il10* gene expression in HT-29 cells (p<0.0005).

### *Propionibacterium freudenreichii* WT, yet not the *P. freudenreichii* Δ*slpB* mutant, improves mucosal preservation in the ileum of mice treated with 5-FU

**Figure 3:**
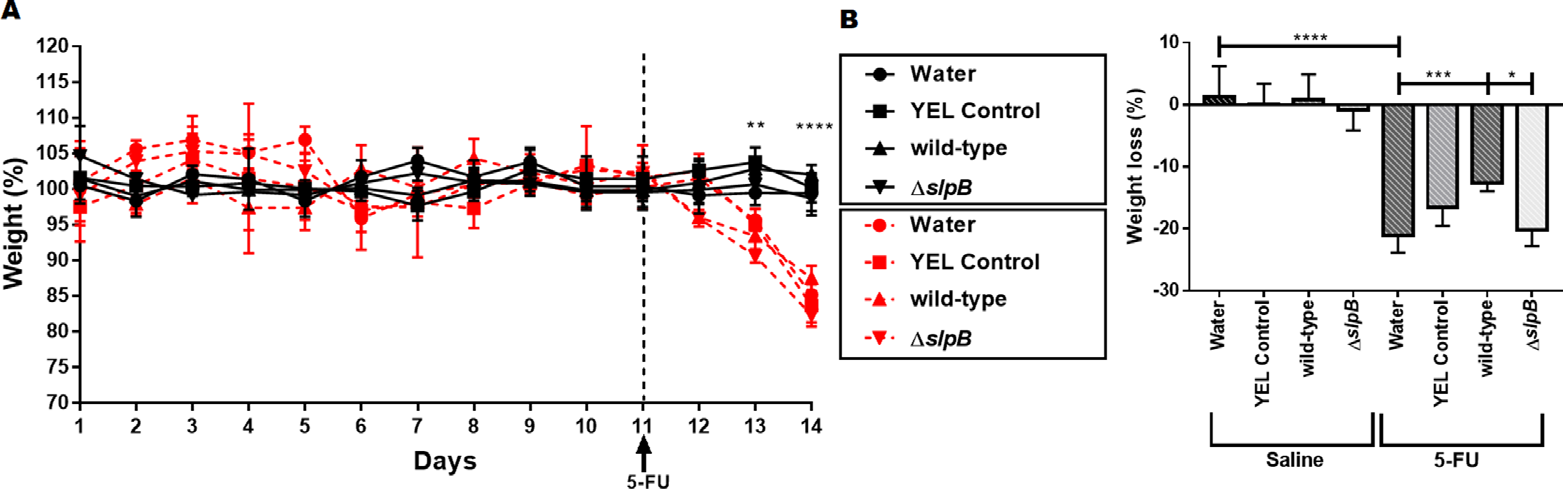
*Propionibacterium freudenreichii* WT strain prevents weight loss in 5-FU-treated mice **(A)** Time-course of body weight for mice receiving YEL culture medium YEL (YEL control), the probiotic strain *P. freudenreichii* 129 WT (wild-type), or the mutant strain *P. freudenreichii* (Δ*slpB*). Black lines correspond to the groups injected with saline *i.p.* and red lines to the groups injected with 5-FU *i.p*. **(B)** Weigh**t** loss observed after 5-FU injection and differences across groups. The means and standard deviations are calculated from daily weighing of 18 animals per group (Three independent replicates with 6 animals per group). Asterisks represent statistically significant differences as follows: ^*^ p <0.05; ^**^ p <0.01; and ^***^ p <0.001.

To further evaluate the protective role of probiotic administration in the context of mucositis, the effect of *P. freudenreichii* on the weight loss of mice after 5-FU administration was studied. The weight of mice belonging to the 8 experimental groups (see Supplementary Figure 2), was monitored before and after 5-FU administration (Figure 3). The body weight in grams as reported in Supplementary Figure 3. No weight difference was observed between groups before 5-FU injection. However, a reduction in weight was clearly observed after 5-FU injection (p<0.0001), when compared to untreated groups (Figure 3A). In this last group, *P. freudenreichii* WT strain consumption significantly limited weight loss: 13%±1.15 (p<0.001), compared to group receiving water: 20.94%±3.21 (Figure 3B). By contrast, the mutant *P. freudenreichii* Δ*slpB* failed to limit weight loss (19.34%±2.58), compared to *P. freudenreichii* WT group, p<0.05).

**Figure 4:**
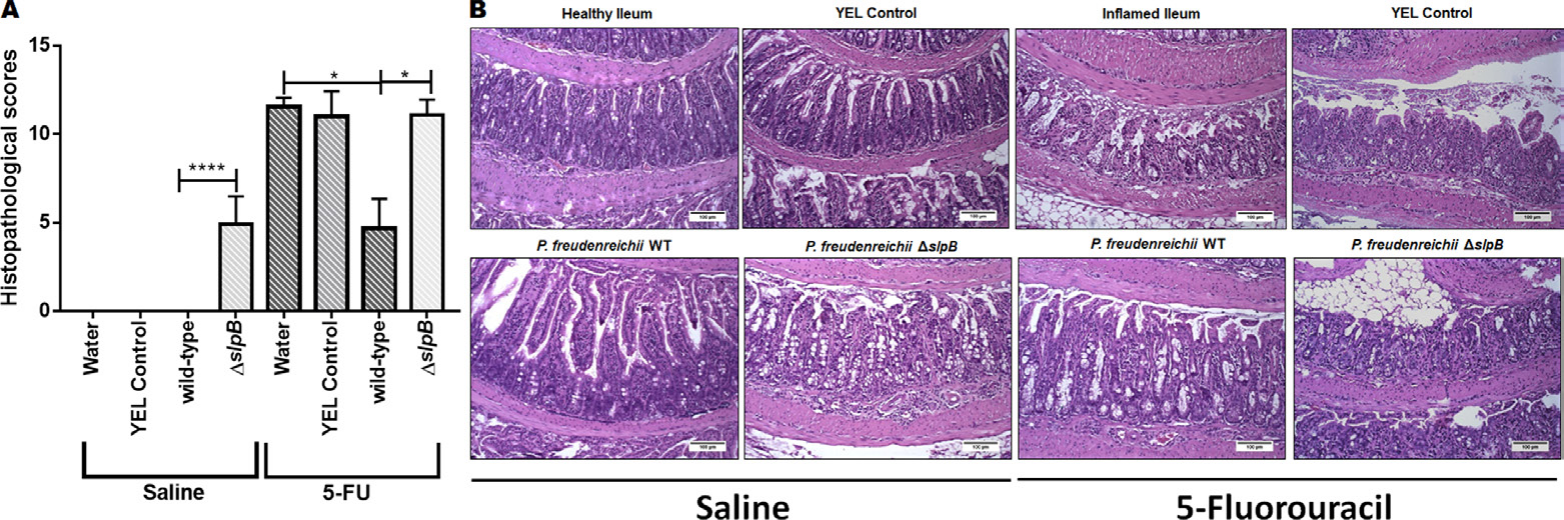
*Propionibacterium freudenreichii* WT strain alleviates mucosal damage in the ileum of 5-FU-treated mice while mutant strain *P. freudenreichii* Δ*slpB* causes inflammation in healthy mice **(A)** Histopathological score obtained in healthy and 5-FU-treated mice. The means and standard deviations are calculated from ileum section of 18 animals per group (Three independent replicates with 6 animals per group). Asterisks represent statistically significant differences as follows: ^*^ p <0.05; ^**^ p <0.01; ^***^ p <0.001; ^****^ p <0.0001. and **(B)** Representative images of H&E-staining of mice ileal mucosa, demonstrating histopathology. The image acquisition was done with objective magnification at 20x. Scale bar=100μm.

Although mucositis may affect the whole digestive tract, we examined damages at the ileal level for all mice, as a well-established readout. Regarding histopathological analysis, control groups injected with saline showed no significant difference in ileum mucosal pattern, whether they consumed water, YEL culture medium, or a YEL culture of *P. freudenreichii* WT (Figure 4A). However, consumption of the mutant strain *P. freudenreichii* Δs*lpB* increased histopathological score (Figure 4A), leading to epithelium flattening, areas of erosion and ulceration in the ileum mucosa (Figure 4B). Moreover, submucosa and muscular layer were thicker than in the other control groups (water, YEL and *P. freudenreichii* WT). In the submucosa layer, vessels were dilated, and edema intense. Some areas presented focal hemorrhage in the muscular layer. Furthermore, infiltration by immune cells, polymorphonuclear and mononuclear cells, was observed. These damages are further evidenced in Supplementary Figure 4.

Mucositis histopathological score translates the clear changes in the morphological structure of the ileum. This includes the intensity of cells infiltrate in the *lamina propria*, changes in mucosal architecture and presence of ulceration. In 5-FU-treated groups consuming water and YEL, this corresponded to increased submucosa and muscular layer, villi shortening, epithelium flattening, increased number of inflammatory cells, with diffuse mononuclear polymorphonuclear inflammatory infiltrate in the *lamina propria* (Figure 4B), when compared to the healthy ileum. Consumption of *P. freudenreichii* WT significantly reduced histopathological scores, compared to control 5-FU-treated groups (water and YEL) p<0.001 (Figure 4A). This corresponded to a reduction in infiltration, in ulceration and in alterations of the intestinal mucosa (Figure 4B, *P. freudenreichii* WT). The mutant *P. freudenreichii* Δ*slpB*, by contrast, failed to alleviate the tissue damages caused by 5-FU (Figure 4B).

**Figure 5:**
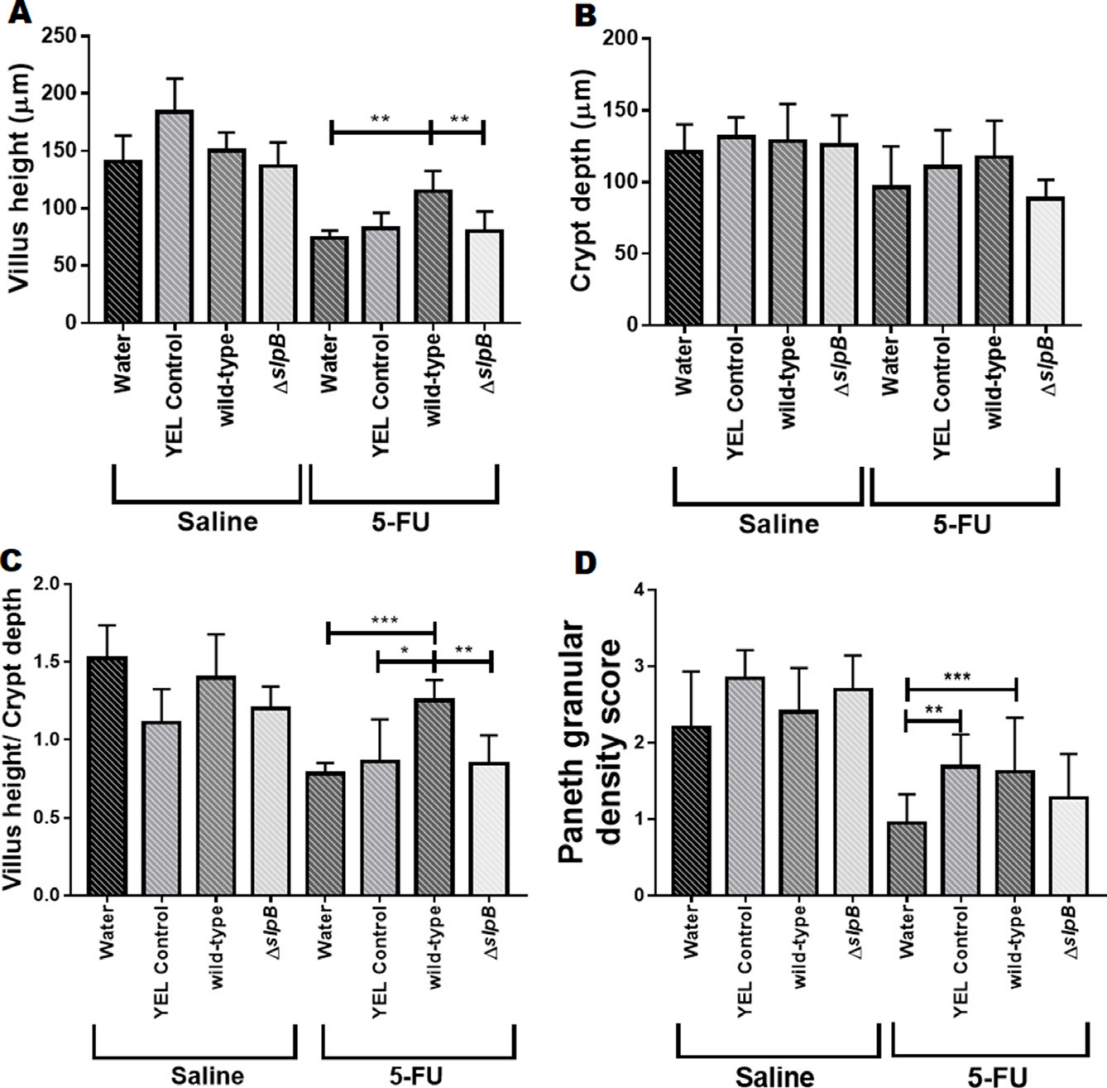
*Propionibacterium freudenreichii* WT strain protects villus architecture and Paneth cells secretory granules density during 5-FU-induced mucositis Morphometric analysis of villus height **(A),** crypt depth **(B)** and ratio villus height/crypt depth **(C)** of mice treated with culture media YEL (control), probiotic strain *P. freudenreichii* WT and mutant strain *P. freudenreichii* Δ*slpB* or without treatment (water) following 5-FU or saline administration. Microscopic morphometric analysis of Paneth cell secretory granules **(D)** of mice treated with culture media YEL (control), probiotic strain *P. freudenreichii* WT and mutant strain *P. freudenreichii* Δ*slpB* or without treatment (water) following 5-FU or saline administration. Values were obtained using objective magnification at 40x by measuring ten random images of the ileum of mice. The means and standard deviations are calculated from ileum section of 18 animals per group (Three independent replicates with 6 animals per group). Asterisks represent statistically significant differences as follows: * p <0.05; ** p <0.01; *** p <0.001; **** p <0.0001.

In addition, we measured the height of the villi and the depth of the crypts (Figure 5). There was no significant difference between the control groups injected with saline. The 5-FU-treated groups showed a reduction in villus height. Consumption of *P. freudenreichii* WT partially restored this height (p>0.0001), compared to groups receiving either water or *P. freudenreichii* Δ*slpB* (Figure 5A). No significant difference was observed between the 5-FU-treated or non-5-FU-treated groups in terms of crypt depth (Figure 5B).

None of the treatments had a significant effect on the granular density of Paneth cells, in the absence of 5-FU. In the context of 5-FU-mucosistis, granular density was reduced. While the mutant strain had no effect on this reduction, both YEL and *P. freudenreichii* WT limited this reduction (Figure 5D).

### *Propionibacterium freudenreichii* WT, yet not the *P. freudenreichii* Δ*slpB* mutant, prevents 5-FU-induced gut permeability

**Figure 6:**
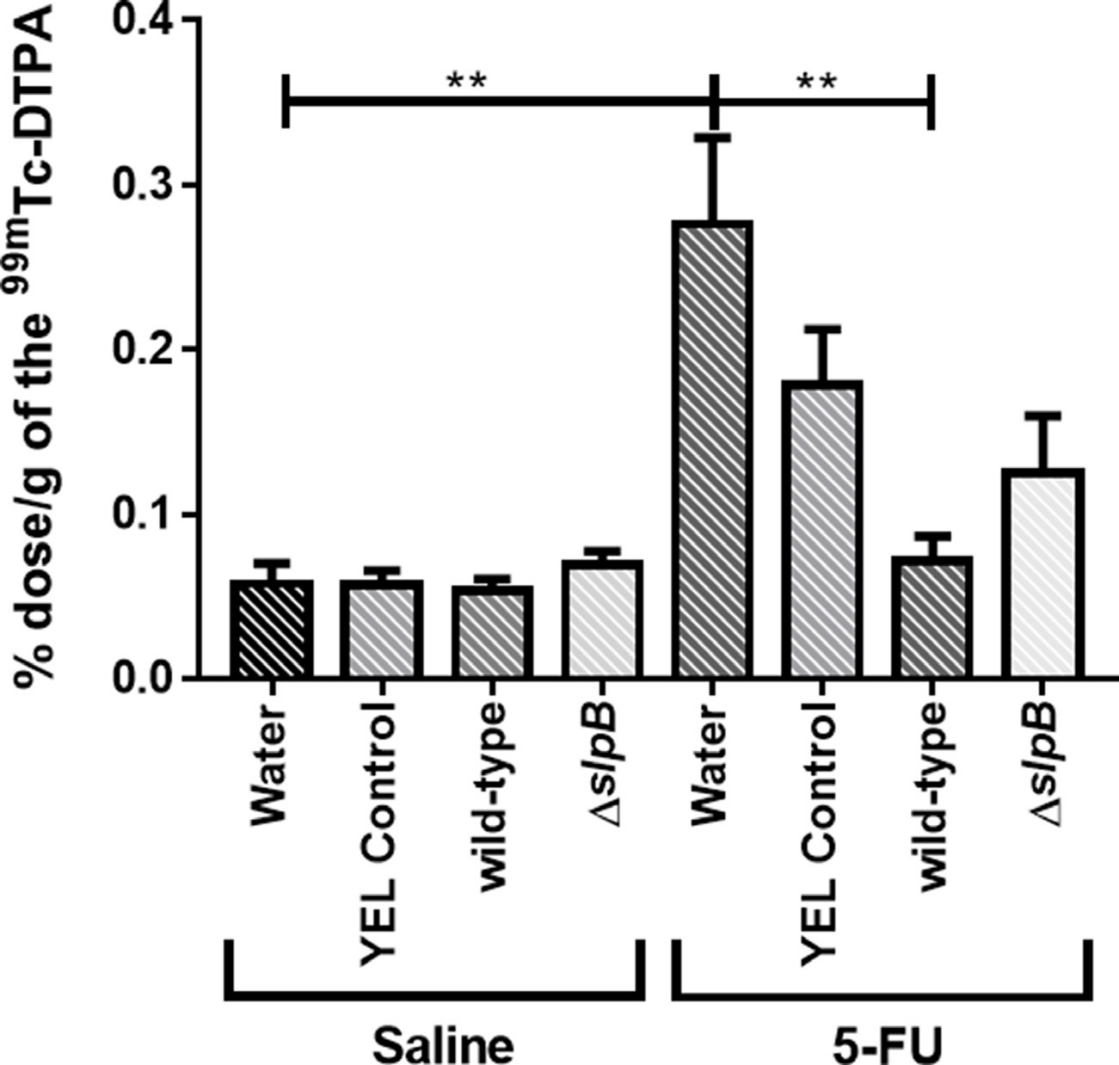
*Propionibacterium freudenreichii* WT consumption decreases intestinal permeability in 5-FU-treated mice Intestinal permeability was measured 72 h after induction of mucositis by radioactivity determination of technetium-99 m (99mTc-DTPA) in mice blood. The means and standard deviations were calculated from one independent experiment for each of the five mice per group. Asterisks represent statistically significant differences between strains and were indicated as follows: *p < 0.05; **p < 0.01; ***p < 0.001, and ****p < 0.0001.

Intestinal permeability was evaluated following oral gavage of mice with radiolabeled diethylenetriaminepentaacetate (^99m^Tc-DTPA) and subsequent quantification of radioactivity in the blood. There was no effect of the different treatments on permeability in control conditions (Figure 6, saline). However, as expected, 5-FU injection significantly increased intestinal permeability, as indicated by ^99m^Tc-DTPA amounts in the mice blood, compared to the control groups (Figure 6, 5-FU water control group). However, the consumption of *P. freudenreichii* WT strain (5-FU wild-type group) significantly prevented (P < 0.01) the 5-FU-induced increase in intestinal permeability. By contrast, consumption of the *P. freudenreichii* mutant strain (5-FU Δ*slpB* group) failed to prevent this induced permeability (p<0.2175).

### *Propionibacterium freudenreichii* Δ*slpB* mutant, yet not the WT strain, induces Th17 cells production in mice spleen

**Figure 7:**
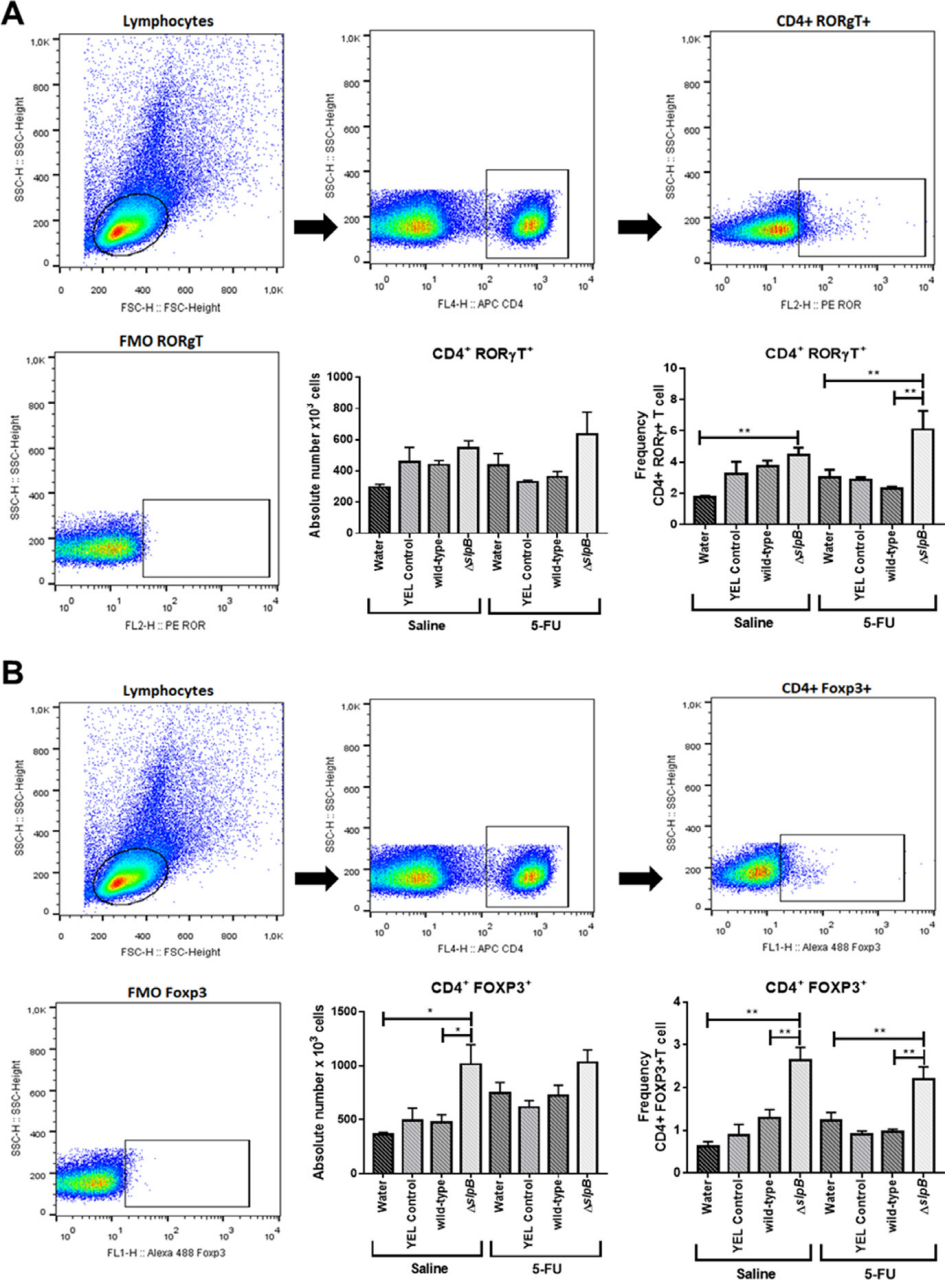
*Propionibacterium freudenreichii* Δ*slpB* mutant strain induces T lymphocyte production in mice spleen after 5-FU-induced mucositis T cells were isolated from mice spleen, and the frequencies of **(A)** CD4+Foxp3+ and **(B)** CD4+ RORγt+ T cells, as frequency (percentage) of CD4+ T cells, were assessed by flow cytometry. The first presented plot represents the gating strategy, based on forward and side scatter, selecting splenocytes as a function of cell size and granularity. Among these, the second presented plot shows gating based on anti-CD4 labeling, selecting T cells. Then, the third presented plot shows representative gated of populations of RORγT **(A)** and FOXP3 **(B)** positive T cells. The fluorescence minus one (FMO) control is shown in the fourth plot. The means and standard deviations were calculated from one independent experiment for each of the five mice per group. Asterisks represent statistically significant differences between strains and were indicated as follows: *p < 0.05; **p < 0.01; ***p < 0.001, and ****p < 0.0001.

T-cell subpopulation was evaluated in mice spleen cells by using flow cytometry. As shown in Figure 7, consumption of the Δ*slpB* mutant strain increased significantly the frequency of both CD4+ ROR-γt+ T (Figure 7A) and CD4+FOXP3+ T (Figure 7B) cells subset in the mice spleen, when compared to water control groups (p>0.01). By contrast, consumption of the WT *P. freudenreichii* strain did not exerted significant effect on neither of these cell subsets in control conditions (p<0.3602 CD4+ ROR-γt+ T and p<0.1613 CD4+FOXP3+). In 5-FU-treated mice, consumption of the Δ*slpB* mutant resulted in a significant increase in CD4+FOXP3+ and CD4+ ROR-γt+ T cells (p<0.0001 and p<0.0367 respectively). However, the frequency of CD4+ ROR-γt+ T cells (Figure 7A) was different between WT and Δ*slpB* groups only in 5-FU-treated mice (p<0.0023), suggesting a boosting effect of Δ*slpB* strain upon inflammatory stimulation.

### *Propionibacterium freudenreichii* reduces secretory IgA production

**Figure 8:**
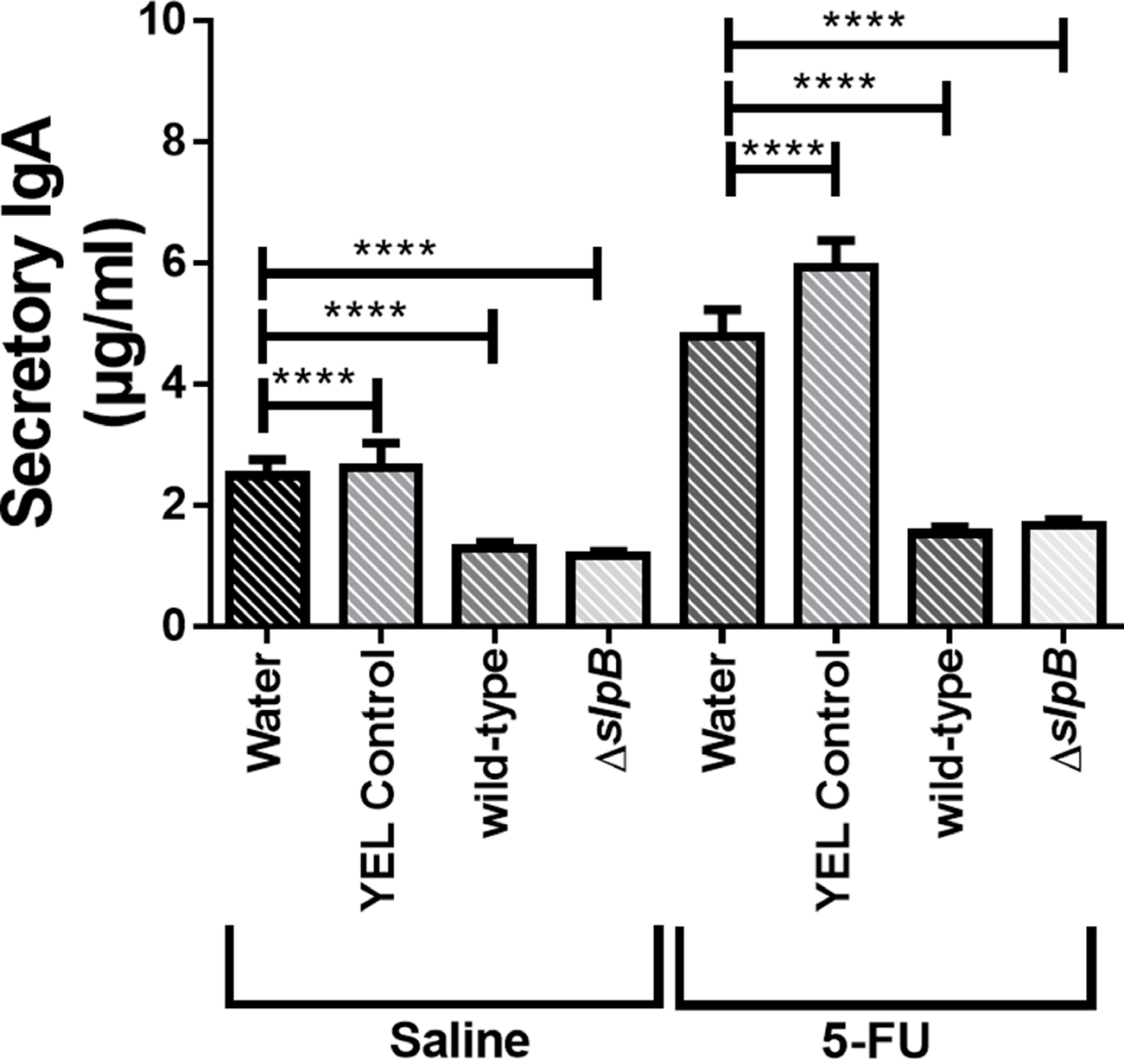
Secretory immunoglobulin A (IgA) in intestinal small bowel content Quantification of immunoglobulin A secretion (sIgA) in the small intestine of healthy or 5-FU-treated mice. The means and standard deviations are calculated from ileum section of 18 animals per group (Three independent replicates with 6 animals per group). Asterisks represent statistically significant differences as follows: ^*^ p <0.05; ^**^ p <0.01; ^***^ p <0.001; ^****^ p <0.000.

Concentration of secretory IgA (SIgA) in the small intestine of mice, 5-FU-treated and non-5-FU-treated, was measured (Figure 8). Injection of 5-FU increased SIgA, in comparison with untreated mice. This induction was totally suppressed by consumption of both strains. Indeed, both *P. freudenreichii* WT and *P. freudenreichii* Δ*slpB* decreased the amount of SIgA in the 5-FU-treated and non-5-FU-treated groups.

### *Propionibacterium freudenreichii* WT and ΔslpB mutant strains differentially modulate gene expression in the mice ileum

**Figure 9:**
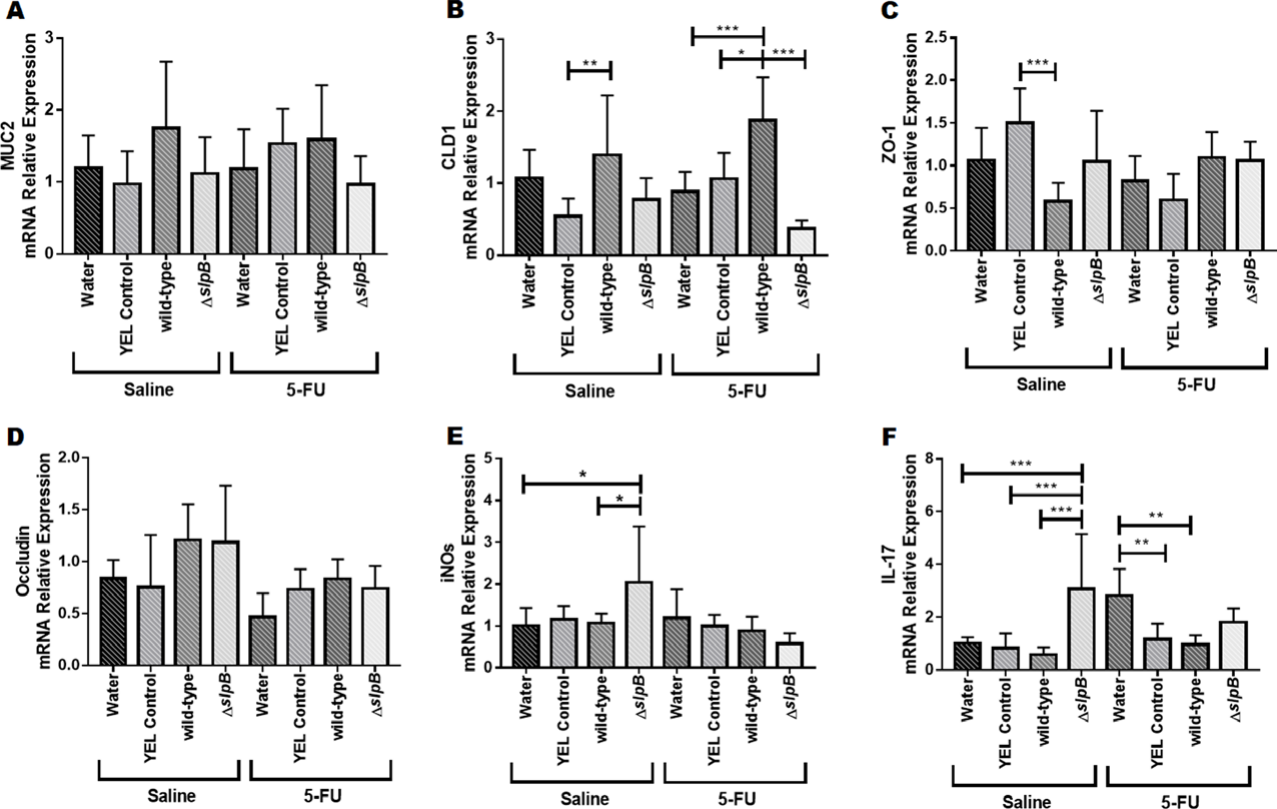
*Propionibacterium freudenreichii* Δ*slpB* induces expression of IL-17 and of inducible NOS (*iNOS*) in healthy mice mRNA relative expression of genes **(A)** *muc2*, **(B)** *cld1*, **(C)** *zo1*, **(D)** *ocln*, **(E)** *iNOS*, and **(F)** *Il17* in mice treated with culture media YEL (control), probiotic strain *P. freudenreichii* WT and mutant strain *P. freudenreichii* Δ*slpB* or without treatment (water) following 5-FU or saline administration. Expression levels was monitored by RT-PCR. The means and standard deviations are calculated from 6 animals per group from 3 independent replicates and each quantification was done in triplicate (technical triplicates). Asterisks represent statistically significant differences between strains and were indicated as follows: *p < 0.05; **p < 0.01; ***p < 0.001, and ****p < 0.0001.

In healthy mice (injected with saline) and in mice injected with 5-FU, no significant difference was found regarding expression of *muc2* gene (Figure 9A). Consumption of *P. freudenreichii* WT significantly increased cld1 gene expression levels in mice injected with 5-FU, compared to water (p<0.001), YEL (p<0.05) or the mutant *P. freudenreichii* Δ*slpB* (p<0.001) (Figure 9B). Consumption of *P. freudenreichii* Δ*slpB* failed to increase *cld1* gene expression. Expression levels of *zo1* only showed significant differences (p<0.001) between YEL and *P. freudenreichii* WT in heathy mice (saline) (Figure 9C). Expression of Occludin (*ocln*) was monitored and no significant difference was found (Figure 9D). Expression of *iNOS* (inducible nitric oxide synthase) was poorly affected, except a trend towards enhanced expression as a result of consumption of the mutant strain, compared to groups receiving water (p<0.05) and *P. freudenreichii* WT (p<0.05), in healthy mice (Saline) (Figure 9E). In addition, consumption of the mutant strain induced expression of *Il17* in healthy mice (p<0.0001) (Figure 9F). In 5-FU-treated mice receiving water, 5-FU triggered a significant induction of *Il17*, which was mitigated by consumption of YEL medium YEL (p<0.01) or of the *P.* freudenreichii WT culture (p<0.01).

### *Propionibacterium freudenreichii* WT and ΔslpB mutant strains differentially modulate cytokines production in the mice ileum

**Figure 10:**
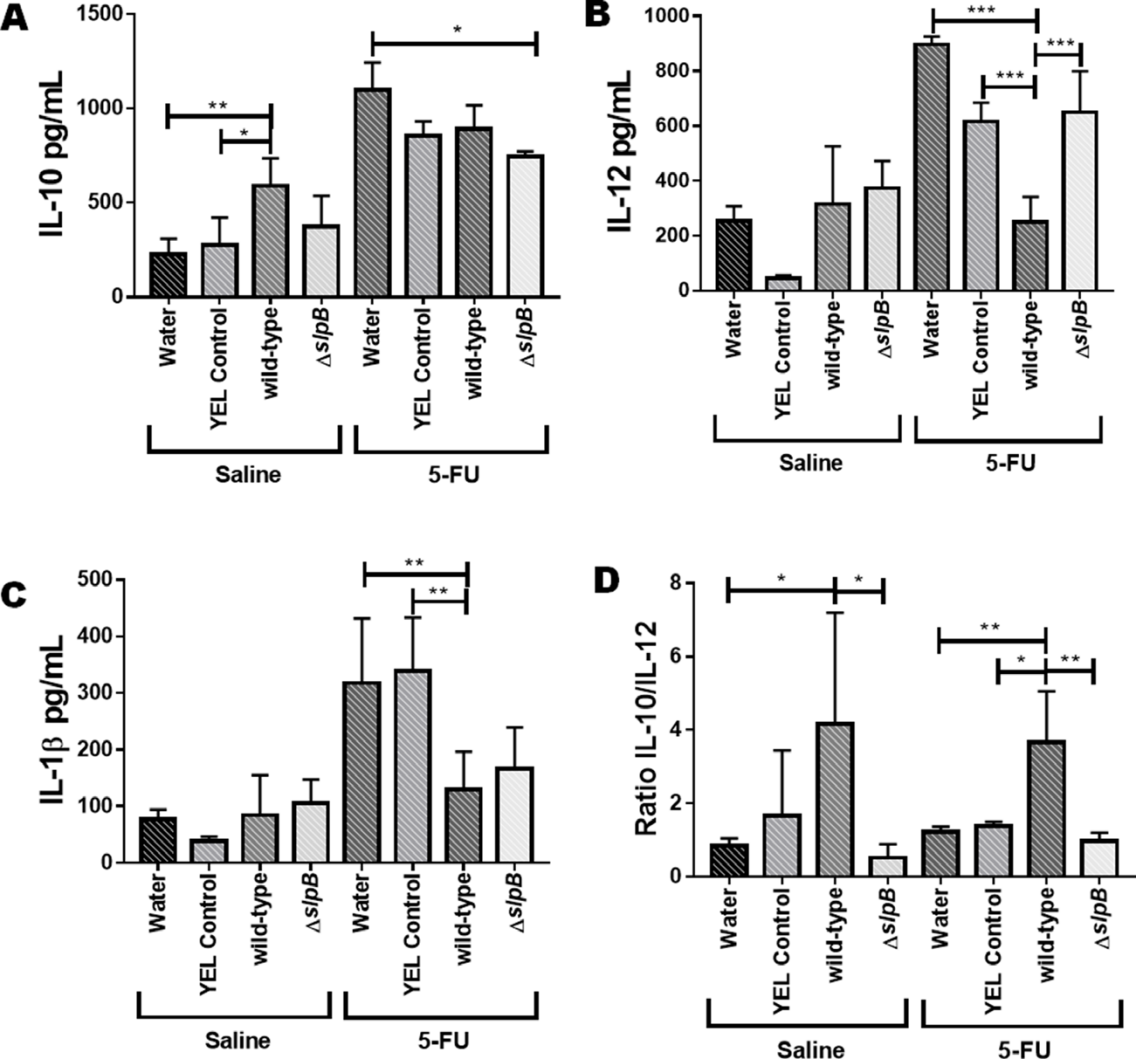
*Propionibacterium freudenreichii* WT strain reduces the pro-inflammatory cytokine IL-12 production during 5-FU-induced mucositis The secreted levels of **(A)** IL-10, **(B)** IL-12, **(C)** IL-1β, and **(D)** IL-10/IL-12 ratio were determined in the supernatant of homogenized mice ileum tissue using ELISA. Mice consumed water, YEL culture medium (YEL control), a YEL culture of the probiotic strain *P. freudenreichii* WT (wild-type) or a YEL culture of the mutant strain (Δ*slpB*). The means and standard deviations are calculated from 6 animals per group from 3 independent replicates and each quantification was done in triplicate (technical triplicates). Asterisks represent statistically significant differences between strains and were indicated as follows: *p < 0.05; **p < 0.01; ***p < 0.001, and ****p < 0.0001.

Cytokines were quantified by ELISA in the intestinal mucosa of the 5-FU-treated (5-FU) and non-5-FU-treated groups (Saline) (Figure 10). In mucositis conditions  (5-FU), the disease drastically induced all the measured cytokines. Consumption of *P. freudenreichii* WT enhanced the ileal IL-10 concentration in healthy mice (p<0.05). However, no significant effect on IL-10 levels was found in mucositis mice (Figure 10A). IL-12 was enhanced by 5-FU and this 5-FU-induction of IL12 was prevented by consumption of *P. freudenreichii* WT (p<0.001), when compared to the YEL medium control, while the mutant failed to do so (Figure 10B). None of the treatments affected IL-1β concentration in control healthy mice. In mucositis mice, 5-FU caused an increase in IL-1β concentration. Consumption of *P. freudenreichii* WT however reduced this 5-FU-induction of IL-1β in the context of mucositis (p<0.01, Figure 10C). Finally, consumption of *P. freudenreichii* WT significantly enhanced the ratio of IL-10 to IL-12 (p<0.01), while the mutant failed to do so (Figure 10D).

## DISCUSSION

The probiotic potential of *Propionibacterium freudenreichii* is based on both the release of beneficial metabolites [1] and on key surface proteins responsible for interactions with the host [12, 13, 23–24]. S-layer proteins form a non-covalently anchored surface-exposed proteinaceous network [25, 26]. They are involved in various processes, such as mediation of the cross-talk with the host [23], which includes immunomodulation [10] and adhesion to host cells in *P. freudenreichii* [11]. Immunomodulation and adhesion, two processes tightly linked [27], require S-layer-associated proteins in *Lactobacilus salivarius* REN [28] and in *Lactobacillus acidophilus* NCFM [29, 30]. In *P. freudenreichii* WT, adhesion to intestinal human cells requires SlpB [11]. Moreover, mutation of the *slpB* gene caused pleotropic effects, impairing surface properties, adhesion and stress tolerance [12]. We therefore investigated the impact of this mutation in the context of human intestinal epithelial cells inflammation.

The present report confirms a key role of SlpB in *P. freudenreichii* probiotic potential. In HT-29 cells, *P. freudenreichii* WT has the ability to induce the release of IL-10, and to reduce that of IL-8 [31]. The ability to induce IL-10 plays a crucial role in the prevention of damage during inflammatory processes [32, 33]. *P. freudenreichii* Δ*slpB* strain was shown here to lose this ability to induce IL-10, probably because 1) of the major modifications of the cell surface properties [12] and 2) of reduced adhesion [11]. Accordingly, purified *P. freudenreichii* SlpB protein induced IL-10 expression in HT-29 cells. Indeed, strains of *P. freudenreichii* which express SlpB induce IL-10 in PBMCs, while those which produce high amounts of SlpA fail to do so [13].

The ability to limit induction of IL-8 is also important in the anti-inflammatory effect, because IL-8 triggers the recruitment of neutrophils in addition to further pro-inflammatory signals in the *lamina propria* [34, 35]. SlpB may also be responsible for the ability to down-regulate IL-8, an anti-inflammatory property shared by several probiotics [36]. Interestingly, the mutant strain, devoid of SlpB, lost the ability to regulate the expression of IL-8, which may decrease their anti-inflammatory potential. This is also observed concerning TNF-α, a pro-inflammatory cytokine, which controls the production of another inflammatory mediator. *P. freudenreichii* WT repressed TNF-α expression in LPS-stimulated HT-29, as reported for other probiotic bacteria or their culture supernatants [37, 38]. Again, the mutant strain *P. freudenreichii* Δ*slpB* was unable to inhibit induction of proinflammatory cytokines.

Extractable surface proteins, including Slps, were already shown to mediate immunomodulation in other probiotic bacteria, including *L. helveticus* MIMLh5 [39], *L. acidophilus* ATCC4356 [40], *L. acidophilus* NCFM [41] and *L. acidophilus* NCK2187 [42]. Other extractable surface proteins are also involved in *L. acidophilus* NCFM and mutation of the corresponding genes can drastically affect immunomodulatory properties [43, 44]. Slps of *L. helveticus* NS8 decreased IL12 induction by LPS in mouse macrophage cell line RAW264.7 [45]. Contrastingly, *L. helveticus* MIMLh5 and its SlpA stimulated the innate immune system by inducing proinflammatory mediators such as TNFα and cyclooxygenase 2 (COX-2) in the human macrophage cell line U937 via TLR2 recognition [39]. Similarly, *L. brevis* Slps induce TNFα in monocyte-derived dendritic cells (moDC) [46].

Toll-like receptors participate in the host cells pro-inflammatory response and play a key role in the regulation of the balance between the Th1 and Th2 type of response [47]. Probiotics’ anti-inflammatory effects may include modulation of Toll-like receptors (TLRs) [48, 49]. Probiotic bacteria may indeed modulate TLRs in a strain-dependent manner. *P. freudenreichii* WT was shown here to enhance TLR2 expression in HT-29 cells, suggesting enhanced reaction towards bacterial LTA, while the mutant failed to do so. In the presence of LPS, no significant effect of *P. freudenreichii*, neither WT nor mutant, was observed regarding TLR2 and TLR9 expression. The pivotal role of the SlpB protein is most evident when monitoring expression of the *tlr4* gene. This expression was shown here to be enhanced by LPS stimulation as previously reported [50]. This induction was totally suppressed by the *P. freudenreichii* wild-type strain, showing another key probiotic ability to impair a pro-inflammatory response machinery. By contrast, LPS-induction of the *tlr4* gene was not repressed by the mutant strain.

Probiotic bacteria may modulate TLRs in a strain-dependent manner. As an example, both *L. plantarum* BFE 1685 and *L. rhamnosus* GG up-regulate TLR2 and TLR9 expression in HT-29 cells [51]. *L. paracasei* F19 strongly induces TLR2 and *E. Coli* K4-induced TLR4 [52]. *L. rhamnosus* GG limits the inflammatory response of porcine intestinal epithelial cells exposed to LPS, by modulating TLR expressions and inhibiting MAPK and NF-κB signaling [53]. By contrast, *Lactobacillus rhamnosus* LGG decreases the expression of TLR2 and TLR-9 in HT-29 cells exposed to *Salmonella* or ot LPS [51, 53]. Each probiotic strain has its specific properties and may specifically modulate expression of pro and anti-inflammatory cytokines and receptors in HIECs.

Considering the above-mentioned *in vitro* regulation of key cytokines and receptors by *P. freudenreichii* WT, expressing SlpB, we decided to exploit such immunomodulatory properties, and to address the importance of SlpB, in a preclinical relevant mucositis model. Mucositis is an inflammatory disease that significantly affects cancer patients undergoing antineoplastic chemotherapy such as 5-fluorouracil (5-FU). Available treatments for mucositis have limitations and probiotics are considered in this context [19]. * P. freudenreichii* WT was able here to reduce the tissue damages caused by 5-FU, to preserve villi height, to limit *lamina propria* infiltration by inflammatory cells and weight loss, in agreement with other studies showing the efficacy of other probiotics [54–56]. We observed such damages at the ileal level, although mucositis may affect the whole digestive tract, as it is a well-established mucositis readout in mice [54–56]. The observed decrease in SIgA levels can be correlated with the protected integrity of the epithelial barrier and consequently protection against pathogens [57–59]. However, mutation of the slpB gene did not change the effect of propionibacteria on sIgA levels. Levels of SIgA increased, when inflammatory stimuli threatened the integrity of the mucosa [58]. They decreased in the group treated with *P. freudenreichii* WT, suggesting that inflammation was contained.

To further evaluate systemic inflammation, we analyzed CD4+T cells expressing FOXP3+ and RORγt+ in mice spleens. In accordance with histology and with cytokines modulation, we observed an amplification of the immune process caused by Δ*slpB* mutant strain, with increased frequencies of both CD4+Foxp3+ and CD4+ROR-γt+ cells in spleens of healthy and 5-FU-treated mice. Regulatory T cells (Tregs) can suppress a wide range of immune cells and play a key role in the maintenance of homeostasis [60], as well as in physiological and pathological immune responses [61]. In our study, the increase in frequency of Tregs might be a compensatory mechanism to counterbalance the cells and mediators of inflammation regarding innate and adaptive cells caused by Δ*slpB* mutant strain.

It is known that CD4+ ROR-γt+ cells are involved in the pathology of inflammatory bowel diseases such as ulcerative colitis and Crohn’s disease [62, 63]. It is plausible that, after Δ*slpB* mutant strain consumption, naïve T cells start expressing RORγt, polarizing towards a proinflammatory response via the Th17/IL-17A pathway. IL-17A can modulate the activation and recruitment of neutrophils in the ileum, which is in accordance with the increased histological score and the enhanced expression of the *Il-17a* gene [64]. In addition, the mutant strain enhanced a subpopulation of CD4+Foxp3+ Treg cells. Regulatory CD4+ T cells expressing Foxp3 are very abundant throughout the intestinal mucosa and their expansion seems to be a homeostatic default mechanism triggered to control the pro-inflammatory Th17 effector cell response triggered by Δ*slpB* mutant strain, as already described in humans [65, 66].

Mucositis induced by 5-FU is linked to an inflammatory process and significantly alters intestinal permeability [67]. We report here that consumption of *Propionibacterium freudenreichii* WT prevented this alteration, a potential that may prevent the exposition of the host to intestinal toxins and bacteria caused by intestinal permeabilization and thus to systemic inflammation [68]. Induced mucositis is accordingly linked to a reduction in the expression of the Claudin-1 gene [69]. Indeed, the structure of tight junctions is an essential factor of the integrity of the epithelial barrier [70, 71]. No significant effect of the different treatments was observed, regarding the expression of genes encoding ZO-1 and Muc2 proteins. However, treatment with probiotic *P. freudenreichii* WT increased *cld1* gene expression in the 5-FU-treated group. Claudin-1 is involved in tight junctions formation and in epithelial cells intercellular adhesion [72]. *P. freudenreichii* WT consumption reduced ileal level of IL-12, which was elevated in the mucositis model [73]. Healthy mice (without 5-FU) consuming *P. freudenreichii* WT (group 1) exhibited enhanced levels of immunomodulatory IL-10, a marker of anti-inflammatory effect also reported for *Lactobacillus acidophilus* [74]. They exhibited a higher IL-10/IL-12 ratio, which was proposed as an anti-inflammatory probiotic effect marker [75]. Moreover, this ratio was also increased in mice with mucositis consuming *P. freudenreichii* (group 7). It is plausible that IL-10 played a key role in *P. freudenreichii* WT containing the inflammatory process driven by 5-FU, given the importance of this cytokine in gut homeostasis.

Mice receiving *P. freudenreichii* Δ*slpB* exhibited a histopathological score different from those receiving the probiotic strain *P. freudenreichii* WT, but closer to that of mucositis control groups. The mutant lost the ability to maintain architectural integrity of the ileum mucosa. In addition to losing its anti-inflammatory capacity, the mutant strain induced inflammation in the ileum of healthy mice (not receiving 5-FU), in accordance with its inefficacy to protect from mucositis. It failed to reduce the abrupt weight loss caused by 5-FU, in contrast with the probiotic strain. The *cld1* expression, decreased in mucositis, was restored by consumption of the probiotic *P. freudenreichii* WT, but not by the mutant, in accordance with its inability to restore gut permeability. Accordingly, extractable surface proteins are associated with the induction of the expression of tight junctions gene encoding Claudin-1, Occludin, JAM-1, and ZO-1 by other probiotics [23]. Moreover, consumption of the mutant increased ileal expression of iNOS, inducible nitric oxide synthase, an enzyme responsible for the generation of cytotoxic and immunoregulatory free radical NO, which is linked to inflammatory processes [76]. Its expression is triggered by IL-17, a pro-inflammatory T cell cytokine [77]. The ileum contains a great number of IL-17 producing cells [78]. Thus, the induction of both iNOS and IL-17 by the mutant may contribute to the onset of inflammation in healthy mice and to its inability to alleviate mucositis induced by 5-FU.

## MATERIALS AND METHODS

### Bacterial strains and culture conditions

The wild-type strain *P. freudenreichii* strain ITGP20, equivalent to CIRM-BIA 129 (*P. freudenreichii* WT), was provided by the CNIEL (Centre National Interprofessionnel de l’Economie Laitière) and maintained by the CIRM-BIA (International Centre for Microbial Resources – Food Associated Bacteria). This strain, as well as the genetically modified *P. freudenreichii* Δ*slpB* strain (*P. freudenreichii* Δ*slpB*) [11], were grown at 30°C in Yeast Extract Lactate (YEL) broth [79]. For the *P. freudenreichii* Δ*slpB* mutant, YEL culture media were supplemented with chloramphenicol (10 μg.mL^-1^). The growth of *P. freudenreichii* was monitored by measuring the optical density at 650 nm (OD_650nm_), as well as by counting colony-forming units (CFUs) in YEL containing 1.5% agar, according to Malik and collaborators [79]. Propionibacteria were then used for animal feeding, or harvested in stationary phase (76 h, 2 x 10^9^ CFU.mL^-1^, determined by plate counts) by centrifugation (8,000 × g, 10 min, 4°C) and washed in PBS, prior to surface protein extraction, or to HT-29 cells challenging.

### Purification of SlpB proteins

A one-litre culture of *P. freudenreichii* strain ITGP20 was prepared as indicated above. Propionibacteria, washed in PBS, were centrifuged (8,000 × g, 10 min, 4°C) and resuspended in 5 M Guanidine hydrochloride (Sigma-Aldrich, St. Louis, MO, USA). After 15 min of incubation, 50°C, bacteria were removed by centrifugation (8,000 × g, 10 min, 20°C). The resulting Guanidine hydrochloride extract contains surface extractable proteins as previously described [10]. The Guanidine hydrochloride extract was concentrated, with elimination of molecules below 30 kDa, by diafiltration using vivaspin 20 30,000 MWCO cells (Sartorius, Stonehouse, Gloucestershire, United Kingdom) and following the provided instructions. The concentrated proteins were washed and recovered in PBS buffer using the same diafiltration cells. The resulting extract was separated by FPLC size-exclusion chromatography. An ÄKTA Purifier 10 system (Amersham Biosciences, Uppsala, Sweden) operating at 0.5 mL/min, equipped with a Superdex 75 10/300 column and a Monitor UV-900 detector operating at 280 nm was used. PBS buffer was used as a mobile phase. The chromatogram and corresponding electrophoretic analysis are shown in Supplementary Figure 1 and Supplementary Figure 5. SlpB, eluted at 11 mL, was then used to stimulate HT-29 cells as described below.

### HT-29 cell challenging

HT-29 cells were routinely grown in T-25 flasks in complete medium DMEMc containing (Dominique Dutscher, Brumath, France) 10% (v/v) fetal calf serum (PAN-Biotech GmbH, Aidenbach, Germany), 100 U/mL penicillin and 100 μg/mL streptomycin sulphate) at 37°C with 5% CO_2_. Trypsin (0.05%)/EDTA (0.2%) (Gibco, Saint Aubin, France) was used to release adherent cells for subculturing. For the experiment, 10^5^ cells were seeded in 12-well plates (1 ml of medium per well) and the growth medium was changed every 2 days. HT-29 cells were grown until complete confluence, 1.10^6^ cells per well in 1  ml volume. Prior to challenging cells, complete medium was replaced with antibiotic-free medium for 3 hours. HT-29 cells were subjected to the different treatments: 7h with 100 ng/mL of Lipopolysaccharide (LPS from E. coli 0111: B4, Sigma), or 7h with *P. freudenreichii* WT, or 7h with *P. freudenreichii* Δ*slpB*. In parallel, cells were also subjected to co-treatments for 7 h: LPS in combination with *P. freudenreichii* WT or *P. freudenreichii* Δ*slpB*, MOI 10 (1 x 10^7^ CFU per well in 1 ml volume). The percentage of HT-29 cells viability after the different stimulation conditions was checked by trypan blue staining and the cells viability was not affected.

### HT-29 cell total RNA isolation and gene expression analysis by qRT-PCR

Cellular RNA was isolated with Trizol reagent (Invitrogen Ambion), and cDNA was synthesized using a qScript cDNA synthesis kit (Quanta Biosciences). Real-time PCR reactions were set up in CFX96 real-time system (Bio-Rad, Marne la Coquette, France). Each PCR reaction was performed in a 16 μL reaction mixture containing 5 μL SYBR Green PCR Master Mix (Biorad), 5 μL of properly diluted cDNA (350 ng of cDNA for all genes), 3 μL mixture of each primer (*actβ* and *gapdh* as housekeeping genes, and *il8*, *tnfa*, *il10*, *ifna*, *muc2*, *zo1*, *tlr9*, *tlr4* and *tlr2*) at 300 nM. The negative controls (with no DNA template, only primer pair, water and SYBR Green PCR Master Mix) for each primer set were included in each run. Amplification was carried out using the following program: 3 minutes at 95°C and 40 cycles of 2 steps consisting of 5 seconds at 95°C and 30 seconds at 60°C. The relative quantification of the mRNA levels of the target genes was determined using CFX Manager Software. The transcript level was normalized to the transcript level of housekeeping genes encoding β-actin (*actβ*) and GAPDH (*gapdh*). Finally, the results are presented as fold change using 2^−ΔΔCT^ method for an unknown sample versus the control (untreated HT-29 cells). The sequences of primers used in this study are listed in Table 1. We followed key genes previously reported to translate immunomodulatory response to probiotics in LPS-stimulated intestinal epithelial cells [31, 37, 51, 53]. Each cell treatment was done on 3 independent cultures (biological triplicates). Each quantification was done in triplicate (technical triplicates). The means and standard deviations are thus calculated from 9 values.

**Table 1:** List of primers used in the *in vitro* study

GENE	PRIMER	SEQUENCE (5′→3′)	PRODUCT SIZE (bp)	Reference
*actβ*	β-actinF	TGG CTG GGT GTT GAA GGT CT	238	[37]
	β-actinR	AGC ACG GCA TCG TCA CCA ACT		
*gapdh*	GapdhF	CAA CGA CCA CTT TGT CAA GC	140	
	GapdhR	TTC CTC TTG TGC TCT TGC TG		
*Il8*	IL-8F	TGG CTC TCT TGG CAG CCT TC	238	
	IL-8R	TGC ACC CAG TTT TCC TTG GG		
*Tnfa*	TNFαF	AGC CCA TGT TGT AGC AAA CC	134	
	TNFαR	TGA GGT ACA GGC CCT CTG AT		
*Il10*	IL-10F	AAA GAA GGC ATG CAC AGC TC	132	
	IL-10R	AAG CAT GTT AGG CAG GTT GC		
*Ifna*	IFNαF	CTG AAA CCA TCC CTG TCC TC	147	
	IFNαR	CAC AGG CTT CCA GGT CAT TC		
*muc2*	MUC2F	CAG CAC CGA TTG CTG AGT TG	140	
	MUC2R	GCT GGT CAT CTC AAT GGC AG		
*zo1*	ZO1F	GAA TGA TGG TTG GTA TGG TGC G	191	[90]
	ZO1R	TCA GAA GTG TGT CTA CTG TCC G		
*tlr9*	TLR9F	GAG CGC AGT GGC AGA CTG GGT G	132	[51]
	TLR9R	CAC AGG TTC TCA AAG AGG GT		
*tlr2*	TLR2F	GCA GAA GCG CTG GGG AAT GG	300	
	TLR2R	GGA TGC CTA CTG GGT GGA GAA		
*tlr4*	TLR4F	GGT GGA AGT TGA ACG AAT GG	119	This study
	TLR4R	CAC TGA GGA CCG ACA CAC C		

Gene identification, forward and reverse oligonucleotide sequences 5’–3’, product size and reference.

### Evaluation of probiotic properties of *P. freudenreichii* WT and *P. freudenreichii* Δ*slpB* to prevent mucositis

### Animals

Conventional female BALB/c mice, between 6 and 8 weeks of age, were obtained at Federal University of Minas Gerais (UFMG–Belo Horizonte, Brazil). These Mice were kept in a temperature-controlled room with *ad libitum* access to water and standard chow diet. The study was approved by the Ethics Committee on Animal Experimentation of the Federal University of Minas Gerais (CEUA-UFMG, Brazil, protocol 366).

### Ethics statement

This project was approved by the Ethics Committee on Animal Use at Federal University of Minas Gerais (CEUA/UFMG) with protocol no. 366/2012, related to the present study is in agreement with the Ethical Principles in Animal Experimentation, and was approved in 11/04/2013.

### Experimental set-up

The experimental set-up is illustrated in Supplementary Figure 1. BALB/c mice were randomly divided into eight groups (6 mice per group). Animals were fed daily orally by 5 mL of water (groups 1 and 5); 5 mL of YEL culture medium (groups 2 and 6) or 5 mL of YEL containg 10^9^ CFU mL^-1^ of either *P. freudenreichii* WT (groups 3 and 7) or *P. freudenreichii* Δ*slpB* (groups 4 and 8) for 10 days. On the eleventh day, the consumption of culture medium and bacteria were discontinued and then all groups received only water. Mucositis was induced on the 11th day of the experimental procedure by a single intraperitoneal injection of 5-fluororacil (300 mg/kg) for groups 5 to 8. An injection of saline (NaCl 0.9%) was used as a control for groups 1 to 4 [57]. Mice were euthanized on the 14th day. *In vivo* assays were performed in biological triplicate.

### Histological analysis

For histomorphological analysis, the distal portion of the mice ileum was collected after the euthanasia and washed with PBS. Afterwards, rolls were prepared and immersed in formaldehyde solution (4%, v/v) for tissue fixation. This material was embedded in paraffin, and a 4 μm section of samples were placed on a glass slide and stained with hematoxylin and eosin (HE). Histological inflammation score was determined as described by MacPherson & Pfeiffer [80], measuring three major histological changes in mucositis disease: (i) intensity of the infiltrate of mononuclear and polymorphonuclear cells in the *lamina propria*, (ii) presence of ulceration and erosion and (iii) alterations in mucosal architecture. The score was given according to the severity of the lesion in the tissues: absent (0), mild (1), moderate (2) and severe (3). For morphometric analysis, ten images of the ileum of each animal were randomly captured and analyzed using ImageJ software (version 1.8.0). Granular density of Paneth cells was determined by measuring the intracellular area occupied by secretory granules [57]. Villi height and crypt depth were measured vertically from the tip of villi to the base of the adjacent crypt. Villus height/crypt depth ratio from the intestinal epithelium was also measured [57].

### Measurement of secretory IgA

For measurement of secretory IgA (sIgA), the small bowel of all euthanized mice were washed using PBS. These materials were vortexed, and centrifuged for 30 min at 850 g at 4° C. Afterwards, the supernatant was transferred to a test tube and used for tested by enzyme-linked immunosorbent assay (ELISA) for IgA concentration as previously described by [57]. The results were expressed as the concentration of sIgA (μg/ml) in intestinal fluid, according to the standard curve.

### Flow cytometry analyses of spleen cell subsets

The method used was previously described by Rocha et al [81]. An amount of 1×10^6^ cells were isolated from spleen and resuspended in PBS-BSA-NaN_3_, pH 7.4 (PBS buffer containing 0.2% BSA (Bovine Serum Albumin) and 0.1% NaN_3_). Then, cell surface antigens were labeled with CD4 Monoclonal Antibody (GK1.5), APC (eBioscience) for 30 min at 4°C. Subsequently, for intracellular staining, cells were fixed and permeabilized with Fixation/Permeabilization working solution (eBioscience) for one hour on ice prior to incubation with Alexa Fluor® 488 Rat Anti-Mouse Foxp3 (BD Pharmingen™) or PE Mouse anti-Mouse RORγt (BD Pharmingen™) for 30 min at 4°C. The cells were then washed in PBS-BSA-NaN_3_ (centrifuged at 1200× g for 5 min at 4°C), and resuspended in 200 μL of the same buffer containing 1% v/v paraformaldehyde. Finally, cells were analyzed using a FACS Calibur cytometer (Becton Dickinson, East Rutherford, NJ, USA) and data was analyzed using the FlowJo software (Tree Star, Ashland, OR, USA). At least 10,000 events were counted for each sample. The gating strategy is based on forward and side scatter, then on anti-CD4 labeling, selecting T cells, then on the selection of RORγT and of FOXP3 (B) positive T cells. Dead cells were excluded by size and lymphocytes were gated using FSC/SSC analysis. Doublets were excluded using FSC channel (height x width). FMO was used to set the threshold for labelling superior to isotype control. CD4+ T cells were then gated as previously described [82, 83]. Among these, either RORγT+ e or FOXP3+ cells were selected.

### Intestinal permeability

On the last experimental day, after 72 hours of mucositis induction, all animals received 0.1 mL diethylenetriaminepentaacetate acid (DTPA), labelled with 18,5 MBq of ^99m^technetium, by gavage. Four hours later, the blood was collected, placed in appropriate tubes for radioactive determination and weighing [67]. Results were calculated as percentage of dose per g of blood, by the following equation: % dose/g blood = (cpm in g of blood/cpm dose of standard) × 100 cpm (counts of radioactivity per minute) [68].

### Intestinal tissue preparation and cytokine quantification by ELISA

For the quantification of cytokines, the ileum were weighed and homogenized in PBS containing 0.05% Tween-20 (Sigma-Aldrich, St. Louis, MO, USA), phenylmethylsulfonyl fluoride 0.1 mM (Sigma- Aldrich, St. Louis, MO, USA), benzethonium chloride 0.1 mM (Sigma-Aldrich, St. Louis, MO, USA), EDTA 10 mM (Synth, São Paulo, São Paulo, Brazil), and aprotinin A 20 KIU (Sigma-Aldrich, St. Louis, MO, USA). Afterwards, this material was homogenized, centrifuged at 3,000 g for 10 min and the supernatants collected for cytokine assay. Plates were coated with purified monoclonal antibodies reactive with cytokines IL- 10, IL-12 p70 and IL-1β/IL-1F2 (R&D Systems, Inc, USA), overnight at 4°C. Then, plate wells were washed, supernatants were added, and plates were again incubated overnight at 4°C. On the third day, biotinylated monoclonal antibodies against cytokines (R&D Systems, Inc, USA) were added on the plates and incubated for 2 h, at room temperature. Colour was developed at room temperature with 100 μl/well of orthophenylenediamine (1 mg/ml) and 0.04% (v/v) H_2_O_2_ substrate in sodium citrate buffer. The reaction was stopped by the addition of 20 μl/well of 2N H_2_SO_4_. The absorbance was measured at 492 nm using a Microplate Reader Model 680 (BIO-RAD).

### Relative expression of cytokines in mice ileum

#### Mice ileum total RNA isolation

Quantitative expression of genes in ileum tissue was measured according to Oliveira and collaborators [84]. First, small fragments (1 cm approximately) of ileum were collected and stored in RNAlater (Ambion, Austin, USA) at −80 °C until RNA extraction. Total RNA was isolated using an RNeasy mini kit (Qiagen, Hilden, Germany) according to the manufacturer’s recommended protocol. Residual genomic DNA was digested and removed using DNase I (Invitrogen, Waltham, MA, USA) treatment. Samples were then treated with Turbo DNA-free Kit® (Ambion), according to manufacturer’s instruction, for DNA removal. cDNA of each sample was produced with High Capacity cDNA Reverse Transcription kit (Applied Biosystems, Foster City, USA), according to its manual instructions.

#### Mice ileum gene expression analysis by qRT-PCR

Quantitative PCR (qPCR) was performed using iTaq universal SYBR green supermix (Biorad, Hercules, CA, USA) and gene specific-primers for *muc2*, Claudin-1 (*cld1*), *Tjp1*, Occludin (*ocln*), *iNOS* and *il-17a* [85–88] as well as housekeeping genes encoding β-actin (*actβ*) and GAPDH (*gapdh*) [85]. Amplification reactions were performed in a final volume of 10 μl, using 5 μl of SYBR green supermix and 10 ng of cDNA. The amplification program consisted of the following steps: 95°C for 30 sec, and 40 cycles of 95°C for 15 sec and 60°C for 30 sec on an ABI PRISM 7900HT Sequence Detection System (Applied Biosystems, Foster City, CA). Expression levels in control group (with no treatment) were used as calibration data. Results are shown graphically as fold changes in gene expression, using the means and standard deviations of target cytokine expression amount (2^−ΔΔCt^) according to Hellemans, Mortier, De Paepe, Speleman, and Vandesompele (2007) [89]. We monitored expression of key genes previously reported to translate severity of intestinal damages in mice [9, 86–88].

### Statistical analyses

The results were reported as the mean ± standard deviation. Parametric data’s were analyzed using One-Way ANOVA followed by the Tukey or Sidak post-test. Non-parametric data’s were analyzed using Kruskal-Wallis data followed by the Dunns post-test. Graphs and statistical analyzes were performed in GraphPad Prism version 7.00 for Windows (GraphPad Software, San Diego, California, U.S.A.). Asterisks represent statistically significant differences between strains and were indicated as follows: ^*^p < 0.05; ^**^p < 0.01; ^***^p < 0.001 and ^****^p < 0.0001.

## CONCLUSIONS

This study confirmed the anti-inflammatory effects of *P. freudenreichii* strain ITGP20, equivalent to CIRM-BIA 129. In the context of induced mucositis, this probiotic reduced inflammation, limited histopathological damages, and restored intestinal permeability. This is important in the context of chemotherapy-induced mucositis, to prevent possible translocation of pathogens and systemic inflammation and infection. This work moreover demonstrated, by *in vitro* and *in vivo* approaches, that the mutation of the extractable surface protein *slpB* gene affects directly the probiotic effects of *P. freudenreichii*. This is mainly evidenced by the fact that *P. freudenreichii* Δ*slpB* loses its ability to regulate pro-inflammatory cytokines in LPS stimulated HT-29 cells, and to alleviate 5-FU induced mucositis. This opens new perspectives for exploring S-layer proteins as possible adjuvants in the treatment of mucositis. Understanding the mechanism responsible for this protective effects deepens the knowledge of Propionibacteria immunomodulatory properties. It opens new perspectives for the utilization of this strain, or of extracted SlpB, in order to alleviate the inflammatory process of mucositis. The clinical guidelines for the management of mucositis recently added a suggestion for the use of probiotics.  *P. freudenreichii*, to our knowledge, received the GRAS status for its use in cheese, for a healthy population. The safety of its consumption by cancer patients with compromised immunity and mucosal barrier should be investigated in this aim. Moreover, metagenomic studies should address the impact on the structure and activity of the gut microbiota, given that selected *P. freudenreichii* strains produce bifidogenic factors and other nutraceutical compounds that may modulate the commensal microbiota.

## SUPPLEMENTARY MATERIALS FIGURES



## Abbreviations

DC-SIGNR1: dendritic cell-specific intercellular adhesion molecule-3-grabbing non-integrin
HIEC: human intestinal epithelial cell
LPS: lipopolysaccharides
PBMC: peripheral blood mononuclear cell
RT-PCR: reverse transcription-polymerase chain reaction
SLAP: S-layer associated protein
SLH: S-layer homology domain
Slp: surface-layer protein
TLR: toll-like receptor.
