# Supplementary material for: Probiotic Propionibacterium freudenreichii requires SlpB protein to mitigate mucositis induced by chemotherapy
Source: Oncotarget. 2019 Dec 31;10(68):7198–219. doi: 10.18632/oncotarget.27319 (PMC6944450; doi:10.18632/oncotarget.27319)
Supplement: Supplementary file 1 [file oncotarget-10-7198-s001.pdf]

Probiotic *Propionibacterium freudenreichii* requires SlpB protein to mitigate mucositis induced by chemotherapy

SUPPLEMENTARY MATERIALS

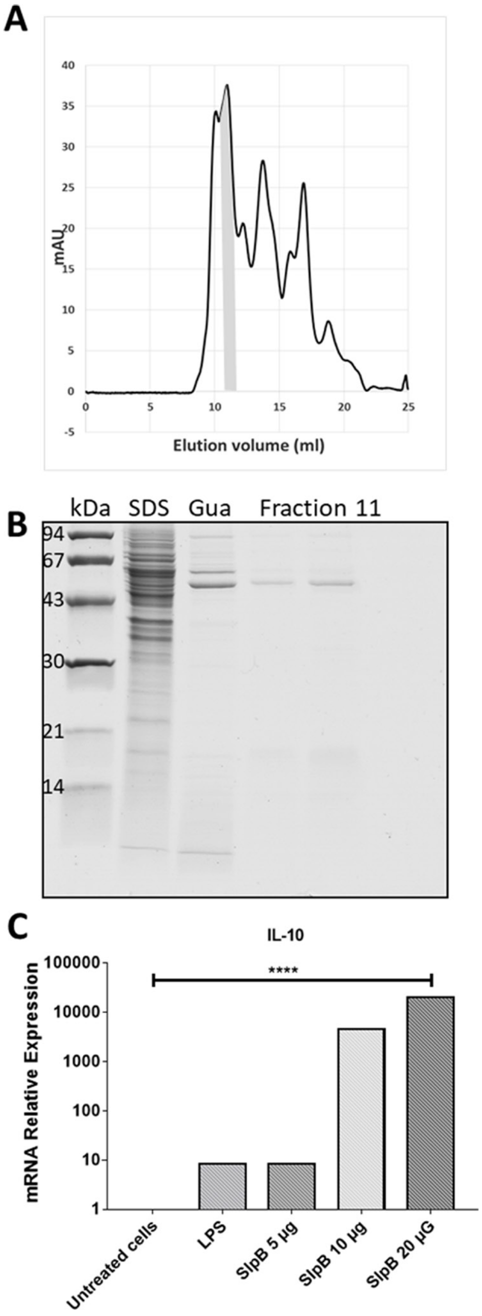

Supplementary Figure 1: Purified SlpB protein induces expression of IL-10 in HT-29 cells. A surface protein Guanidine extract was subjected to size-exclusion chromatography and the chromatogram is shown **(A).** SDS-PAGE was used to analyze the protein content of a whole-cell protein SDS extract, of the Guanidine extract, and of purfified SlpB in fraction 11 **(B)**. SlpB was used to treat HT-29 cells and expression of *il10* was monitored after 7 h of treatment with different doses **(C)**. ***: $p < 0.001$.

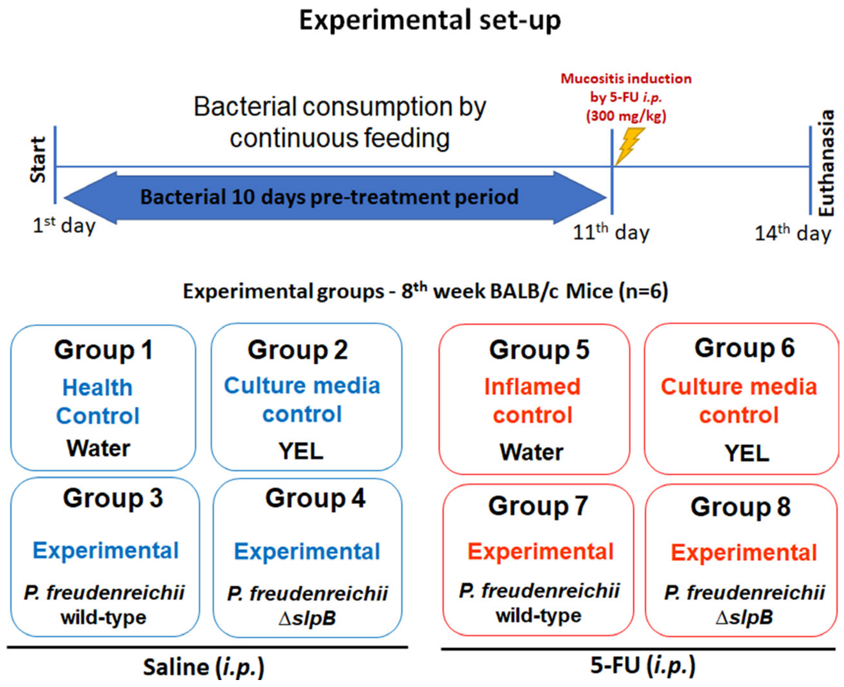

Supplementary Figure 2: Experimental set-up of the animal study. Mice were divided into 8 groups of 6 mice**.** They were given different treatments, as indicated, during 10 days, prior to mucositis induction. Then groups 1 to 4 were injected with a saline solution, while groups 5 to 8 were injected with a solution of 5-FU.

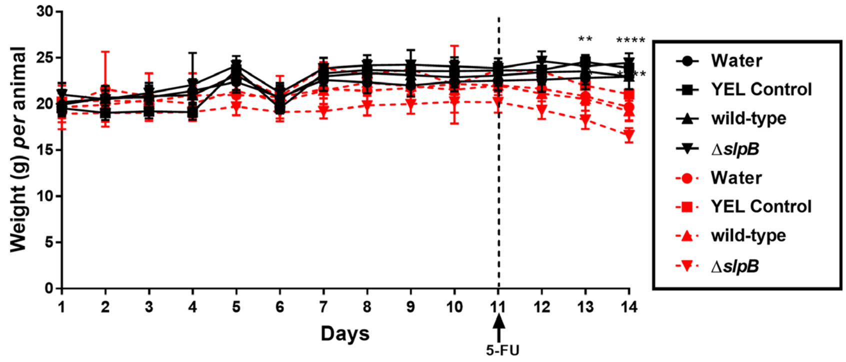

Supplementary Figure 3: Time-course of body weight in grams. Mice receiving YEL culture medium YEL (YEL control), the probiotic strain *P. freudenreichii* 129 WT (wild-type), or the mutant strain *P. freudenreichii* (Δ*slpB*). Black lines correspond to the groups injected with saline *i.p.* and red lines to the groups injected with 5-FU *i.p.*

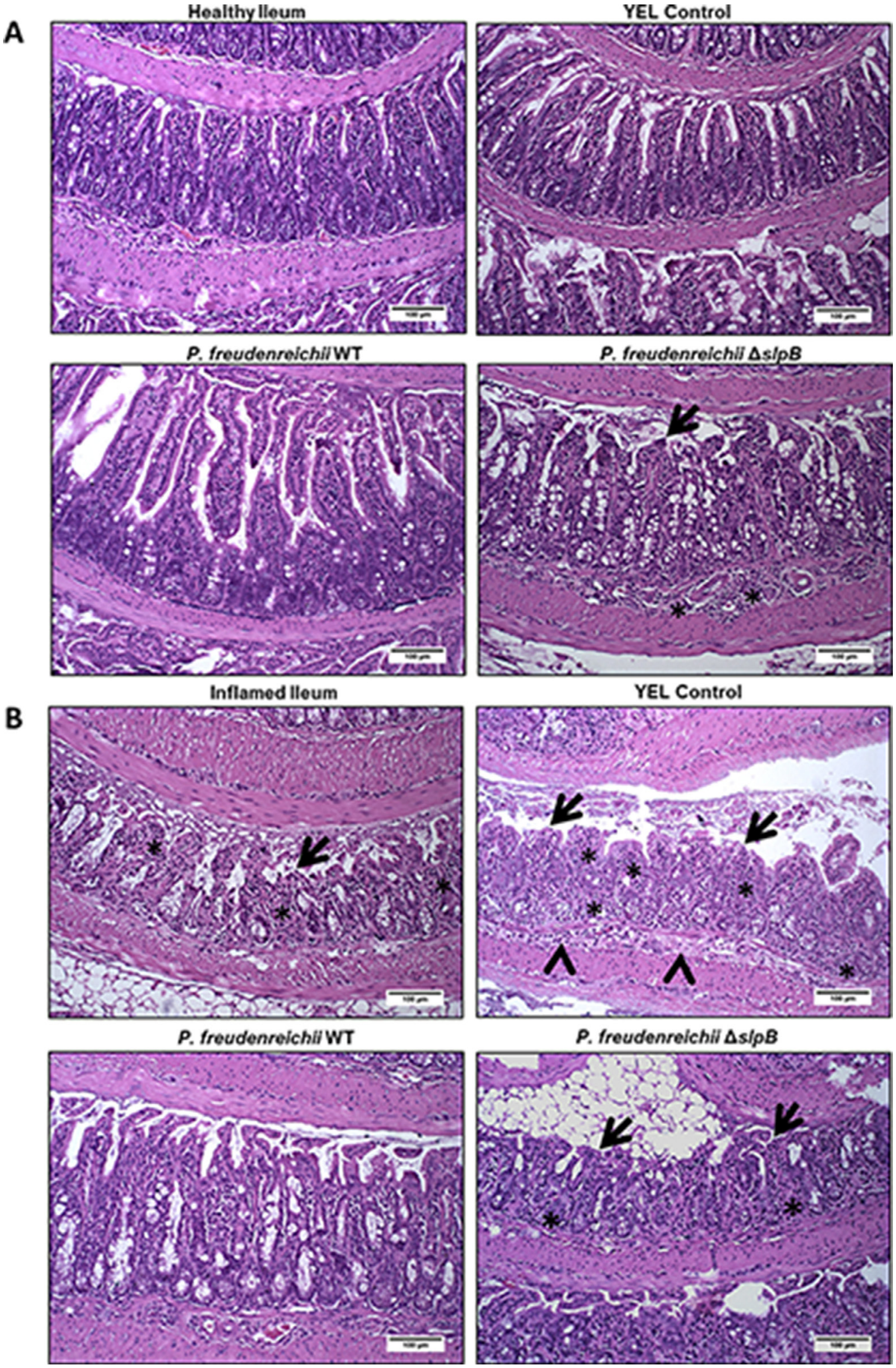

Supplementary Figure 4: Representative images of H&E-stained sections of mice ileal mucosa, demonstrating histopathology. (A) Non inflamed groups and **(B)** 5-FU inflamed groups**.** The image acquisition was done with objective magnification at 20x. Scale bar=100µm. Cellular infiltration was indicated by asterisks (*), Epithelial erosion was indicated by arrows (→), Edema was indicated by arrowheads (>).

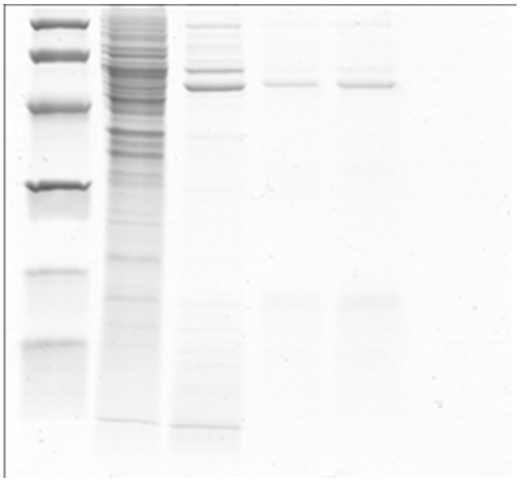

Supplementary Figure 5: Uncropped gel from Supplementary Figure 2.
